# Association of medicaid expansion with lung cancer–specific and overall mortality: A difference-in-differences analysis

**DOI:** 10.1371/journal.pone.0332292

**Published:** 2026-04-09

**Authors:** Oluwasegun Akinyemi, Mojisola Fasokun, Akachukwu Eze, Nkemdirim Ugochukwu, Sumaiyya Arshad, Orimisan Belie, Kakra Hughes, Edward Cornwell III, Gal Levy

**Affiliations:** 1 The Clive O Callender Outcomes Research Center, Howard University College of Medicine, Washington District of Columbia, United States of America; 2 Department of Epidemiology, University of Alabama at Birmingham, Alabama, United States of America; 3 Department of Surgery, Howard University College of Medicine, Washington District of Columbia, United States of America; 4 Department of Oncology and Tumor Biology, Georgetown University, Washington District of Columbia, United States of America; University of Miami, UNITED STATES OF AMERICA

## Abstract

**Introduction:**

Medicaid expansion under the Affordable Care Act (ACA) sought to improve access to timely cancer diagnosis and treatment among low‑income populations. Lung cancer remains the leading cause of cancer-related mortality in the United States, and disparities in outcomes may be sensitive to shifts in insurance coverage. This study evaluates the association between Medicaid expansion and both cancer-specific mortality and overall mortality among adults with lung cancer in California (expansion state) compared with Texas (non-expansion state).

**Methods:**

We conducted a retrospective cohort study using SEER registry data (2007–2021) including adults aged 18–64 years diagnosed with lung cancer. The study periods were categorized as pre-ACA (2007–2013), washout year (2014), and post-ACA (2015–2021). Difference‑in‑differences (DiD) Cox proportional hazards models estimated the change in the hazard of cancer‑specific and overall mortality in California relative to Texas after Medicaid expansion, adjusting for age, sex, race/ethnicity, stage, county‑level income, and treatment. Subgroup analyses evaluated heterogeneity by race/ethnicity, disease stage, income, and treatment modality.

**Results:**

Among 119,937 individuals with lung cancer, 52.1% resided in California and 47.8% in Texas; sample distribution remained similar across pre‑ and post‑ACA periods. Medicaid expansion was associated with an 11.9% reduction in the hazard of cancer‑specific mortality (DiD HR 0.88; 95% CI, 0.85–0.91) and an 11.4% reduction in the hazard of overall mortality (DiD HR 0.89; 95% CI, 0.86–0.91). Mortality reductions varied across subgroups. Significant improvements were observed among White, Hispanic, and Asian/Pacific Islander patients, while no statistically significant change occurred among Black patients. Greater reductions in the hazard of death were seen among patients with distant-stage disease and those residing in higher‑income areas (≥$65,000). Treatment-stratified models showed decreases in mortality among individuals receiving surgery (10.2% reduction) and chemotherapy (8.4% reduction).

**Conclusion:**

Medicaid expansion was associated with meaningful reductions in lung cancer mortality in California relative to Texas, with benefits concentrated among several racial and clinical subgroups. Persistent null effects among Black patients highlight inequities that insurance expansion alone does not eliminate.

## Introduction

Lung cancer remains a significant public health challenge in the United States, with an estimated 238,340 new cases diagnosed and 127,070 deaths expected in 2023 [1–3]. It is the second most common cancer among both men and women, yet it accounts for the highest number of cancer related deaths, surpassing breast, prostate, and colorectal cancers combined [3,4]. The burden of lung cancer mortality is particularly striking, with men exhibiting slightly higher death rates than women [5,6]. This disease not only claims lives but also imposes a substantial financial toll on the U.S. healthcare system, with direct medical costs exceeding $13 billion annually, which includes the initial phase and continuing care phase [7]. Beyond the healthcare expenses, lung cancer results in significant indirect costs, including billions of dollars in lost productivity due to premature deaths and work absences, highlighting the extensive economic impact of this disease on society [8–10].

Social determinants of health (SDOH) play a critical role in influencing lung cancer outcomes [11–13]. Disparities in mortality rates are pronounced across racial, ethnic, and socioeconomic lines, with the highest burden observed among Black, Hispanic, and low-income populations [14,15]. Socioeconomically disadvantaged individuals face barriers to early detection and access to timely, effective treatment, exacerbating poor outcomes [15]. These disparities underscore the intersection of healthcare access, income inequality, and systemic racism in shaping health inequities, particularly for lung cancer, a disease with significant morbidity and mortality.

The Affordable Care Act (ACA), signed into law in 2010, sought to address these disparities through sweeping healthcare reforms aimed at increasing insurance coverage, reducing healthcare costs, and improving population health [16,17]. One of the ACA’s major components is Medicaid expansion, a policy designed to extend health coverage to low-income adults [18]. The staggered implementation of Medicaid expansion across states provides a natural experiment to assess its impact on health outcomes. By reducing financial barriers and increasing access to preventive services and timely treatment, Medicaid expansion has the potential to improve outcomes for cancers such as lung cancer, which require early and continuous care for optimal management.

In California, Medicaid expansion, implemented in 2014 under the Medi-Cal program, aimed to increase healthcare access among low-income residents. The state’s decision to expand Medicaid was part of a broader strategy to address healthcare inequities and improve outcomes for its diverse population [19]. Studies from states that adopted Medicaid expansion have reported reductions in cancer-related mortality, increased early-stage diagnoses, and improved access to treatment [20,21]. These findings suggest that Medicaid expansion may play a crucial role in mitigating the burden of lung cancer, particularly among underserved populations.

This study aims to evaluate the impact of Medicaid expansion on lung cancer outcomes in California compared to Texas, a non-expansion state. By examining differences in cancer specific and overall mortality between these states, this study seeks to provide insights into the role of Medicaid expansion in reducing disparities and improving survival for individuals diagnosed with lung cancer.

## Methodology

### Study design and data source

We conducted a retrospective cohort study using the Surveillance, Epidemiology, and End Results (SEER) cancer registry, a population-based dataset that captures incident cancers, treatment patterns, and vital status [22]. The specific SEER research version utilized for this study contained complete, analyzable lung cancer data for only a limited number of states with consistent longitudinal coverage across the study period. Within this dataset, California was the largest Medicaid expansion state, while Texas was one of only two non-expansion states with complete and continuous data availability.

Given that the available non-expansion states were few and not representative of all U.S. non-expansion states, pooling them to construct a broader non-expansion comparison group could introduce heterogeneity and bias. Therefore, to preserve internal validity and ensure a clear policy contrast, we limited the analysis to California and Texas, two of the largest U.S. states with comparable population size, robust cancer registry data, and distinct Medicaid expansion policies—California having expanded Medicaid in 2014 and Texas remaining a non-expansion state throughout the study period [23]. California, the largest SEER expansion state with continuous annual data linkage, implemented Medicaid expansion in 2014 through Medi-Cal, substantially broadening healthcare access and reducing uninsured rates [24]. In contrast, Texas one of the only large non-expansion states with complete SEER data did not adopt Medicaid expansion during the study period, maintaining persistently higher uninsured rates and more limited access to care [25]. These sharply divergent policy environments, combined with the SEER dataset’s restriction to these two fully represented states, provided a strong, natural quasi-experimental setting for evaluating the association between Medicaid expansion and lung cancer mortality outcomes using a difference-in-differences framework.

### Study population

We included individuals aged 18–64 years diagnosed with lung cancer between January 1, 2007, and December 31, 2021. Patients older than 64 years were excluded to minimize crossover with universal Medicare eligibility.

### Exposure definition and study periods

The exposure of interest was Medicaid expansion under the ACA. California was coded as the expansion state and Texas as the non‑expansion state. The pre‑expansion period was defined as 2007–2013, and the post‑expansion period as 2015–2021. A wash‑in year (2014) was excluded to allow full implementation of the policy in California. The interaction between state (California vs. Texas) and time period (pre‑ vs. post‑expansion) provided the difference‑in‑differences (DiD) estimate of the policy’s association with outcomes.

### Outcomes and time scale

The primary outcomes were cancer-specific mortality (CSM) and overall mortality (OM), as reported in the SEER registry. SEER provides the date of cancer diagnosis and the date and cause of death, enabling construction of time-to-event variables. Survival time was calculated in years from the date of diagnosis to the date of death or censoring at last known follow-up. Because SEER reports mortality outcomes rather than survival probabilities, all analyses were conducted on the hazard of death, and results are presented as hazard ratios (HRs) reflecting the relative change in mortality risk. CSM captured deaths attributed to lung cancer, whereas OM captured deaths from any cause. For both outcomes, individuals who were alive at last contact were censored on that date. Median follow-up time and censoring distributions were summarized to describe completeness of follow-up across states and study periods. Throughout the manuscript, we use terminology specific to mortality “hazard of death,” “mortality risk,” and “change in hazard” to avoid any conflation between mortality and survival.

### Covariates

Covariates included age, sex, race/ethnicity, metropolitan residence, treatment modalities (surgery, chemotherapy, radiation), and disease stage (localized, regional, distant). The SEER version used for analysis reported disease stage using the Combined Summary Stage classification (localized, regional, distant), which ensured consistent staging across years. Income was defined as county‑level mean household income, consistent with SEER’s income reporting structure. Income was dichotomized as <$65,000 vs. ≥ $65,000.

### Missing data

Missing data were minimal across all analytic variables (<0.5%). Accordingly, we performed a complete-case analysis. Given the negligible extent of missingness, this approach is unlikely to introduce meaningful selection bias or materially affect effect estimates; therefore, additional sensitivity analyses using imputation were not pursued.

### Statistical analysis

Descriptive characteristics of patients in California and Texas were compared across the pre- and post–Affordable Care Act (ACA) periods using appropriate univariate tests. Categorical variables were summarized using frequencies and percentages and compared using χ² tests, whereas continuous variables were assessed for normality and compared using two-sample t-tests or Welch’s t-tests when variances were unequal. These descriptive comparisons correspond to the variables presented in Table 1.

**Table 1:** Baseline Demographic and Clinical Characteristics of Patients with Lung Cancer in Texas and California Pre- and Post-ACA Implementation.

Variables (N = 119,937)	Pre-ACA (n = 60,010)			Post-ACA (n = 59,927)		
	Texas (46.9%)	California (53.1%)	P-value	Texas (48.8%)	California (51.2%)	*p*
Age, year, Mean ± SD	56.5 ± 6.5	56.6 ± 6.8	0.05	57.4 ± 6.3	57.3 ± 6.7	0.01
Age(years)			<0.01			0.038
18-45yrs.	1,351 (4.8%)	1,738 (5.5%)		1,390 (4.8%)	1,635 (5.3%)	
45-64yrs.	26,839 (95.2%)	30,082 (94.5%)		27,836 (95.2%)	29,066 (94.7%)	
Sex Male Female			<0.01			<0.01
	15,757 (55.9%)	16,699 (52.5%)		15,276 (53.0%)	15,331 (50.2%)	
	12,433 (44.1%)	15,121 (47.5%)		13,950 (47.1%)	15,370 (49.8%)	
Race/Ethnicity			< 0.01			< 0.01
White	19,575 (69.4%)	20,119 (63.2%)		19,371 (66.3%)	17,106 (55.7%)	
Black	4,591 (16.3%)	3,550 (11.2%)		4,698 (16.1%)	3,198 (10.4%)	
Hispanic	3,330 (11.8%)	3,938 (12.4%)		4,013 (13.7%)	4,733 (15.4%)	
Asian or Pacific Islanders	575 (2.0%)	3,967 (12.5%)		988 (3.4%)	5,215 (17.0%)	
Naive American	95 (0.4%)	201 (0.6%)		117 (0.4%)	264 (0.9%)	
Other	24 (0.1%)	45 (0.3%)		39 (0.1%)	185 (0.6%)	
Grade			< 0.01			< 0.01
Localized	4,274 (16.7%)	4,848 (15.8%)		5,356 (19.8%)	6,087 (20.5%)	
Regional	6,267 (24.5%)	6,544 (21.3%)		6,427 (23.7%)	5,881 (19.8%)	
Distant	15,093 (58.9%)	19,314 (62.9%)		15,329 (56.5%)	17,720 (59.7%)	
Metropolitan Status			<0.01			<0.01
Metropolitans Areas	23,587 (83.7%)	30,553 (96.0%)		24,378 (83.4%)	29,442 (95.9%)	
Rural Areas	4,603 (16.3%)	1,267 (4.0%)		4,847 (16.6%)	1,259 (4.1%)	
Household Median Income			< 0.01			< 0.01
<65K	14,708 (52.2%)	4,508 (14.2%)		10,951 (37.5%)	3,411 (11.1%)	
≥65K	13,482 (47.8%)	27,312 (85.8%)		18,274 (62.5%)	27,290 (88.9%)	
Recommendations			<0.01			< 0.01
Yes, n (%)	6,449 (25.1%)	8,552 (27.3%)		5,873 (22.3%)	8,276 (27.3%)	
No, n (%)	19,282 (74.9%)	22,801 (72.7%)		20,441 (77.7%)	22,022 (72.7%)	
Chemotherapy			<0.01			<0.01
Yes, n (%)	12,960 (46.0%)	16,929 (53.2%)		12,724 (43.5%)	15,745 (51.3%)	
No, n (%)	15,230 (54.0%)	14,891 (46.8%)		16,502 (56.5%)	14,956 (48.7%)	
Treatment			<0.01			<0.01
Chemoradiation	221(0.8%)	466 (1.5%)		261 (0.9%)	390 (1.3%)	
Surgery Alone	25,280 (90.3%)	26,625 (84.5%)		25,461 (87.8%)	25,229 (83.0%)	
Adjuvant Chemotherapy	2,151 (7.7%)	3,874 (12.3%)		2,909 (10.0%)	4,338 (14.3%)	
Surgery & Radiation	337 (1.2%)	536 (1.7%)		380 (1.3%)	436 (1.4%)	
Mortality	20,405 (73.8%)	23,385 (74.4%)	0.11	16,166 (55.7%)	16,064 (52.8%)	<0.01
5- Year Survival (OS)	7836 (22.6%)	8927 (24.2%)	0.77	5693 (27.7%)	6051 (32.4%)	<0.01
5-Year Survival (CSS)	6442 (28.0%)	7643 (27.7%)	0.48	4541 (33.9%)	5046 (38.0%)	<0.01

Table summarizes the baseline characteristics of cancer patients from California and Texas pre- and post-ACA period. The result shows statistically significant differences in sex, race/ethnicity, household income, and metropolitan status.

To evaluate the association between Medicaid expansion and mortality outcomes, we used Cox proportional hazards regression models specified within a DiD framework. The model included main effects for state (California vs Texas), time period (pre-ACA vs post-ACA), and their interaction; the State × Period interaction term represented the DiD estimate and was interpreted as the differential change in the hazard of death in California relative to Texas following Medicaid expansion. Hazard ratios (HRs) and 95% confidence intervals (CIs) were reported, and the percent change in hazard was calculated as (HR − 1) × 100.

All Cox DiD models were adjusted for prespecified covariates, including age, sex, race and ethnicity, disease stage at diagnosis, county-level median household income, and treatment variables (surgery and chemotherapy). Effect modification was assessed using three-way interaction terms (State × Period × subgroup) for race and ethnicity, disease stage, neighborhood income, and treatment type. Subgroup-specific DiD effects were generated using model-derived marginal contrasts, and heterogeneity across subgroups was evaluated using joint Wald χ² tests.

The proportional hazards assumption was assessed using Schoenfeld residuals, with no major violations detected. All analyses were two-sided with α = 0.05 and performed using Stata/SE version 16 (StataCorp LLC).

## Result

Table 1 summarizes the baseline demographic and clinical characteristics of 119,937 lung cancer patients, with 47.87% from Texas and 52.12% from California. The population was further divided into pre-ACA (n = 60,010; 50.0%) and post-ACA (n = 59,927; 49.9%) periods. PreACA, 46.9% of patients were from Texas, and 53.1% were from California; similar proportions were observed post-ACA (Table 1).

Pre-ACA, significant differences were noted between the two states in age, sex, race/ethnicity, household income, and metropolitan status. California had a higher proportion of younger patients aged 18–45 years (5.5% vs. 4.8%, p < 0.01), females (47.5% vs. 44.1%, p < 0.01), and individuals from racial/ethnic minority groups, including Hispanic (12.4% vs. 11.8%, p < 0.01) and Asian/Pacific Islanders (12.5% vs. 2.0%, p < 0.01). A greater percentage of patients in California resided in metropolitan areas (96.0% vs. 83.7%, p < 0.01) and had a household income ≥ $65K (85.8% vs. 47.8%, p < 0.01).

Post-ACA, some disparities persisted, but key improvements were noted in both states. The proportion of individuals with distant disease increased in both states but remained marginally higher in California (59.7% vs. 56.5%, p < 0.01). California patients had high rates of chemotherapy (51.3% vs. 43.5%, p < 0.01) and adjuvant chemotherapy (14.3% vs. 10.0%, p < 0.01) compared to their counterparts in Texas. Notably, five-year overall survival (OS) and cancer-specific survival (CSS) improved in both states, with greater gains in California (OS: 32.4% vs. 27.7%, p < 0.01; CSS: 38.0% vs. 33.9%, p < 0.01) (Table 1).

### Cancer-specific mortality by race/ethnicity

In DID models evaluating CSM, Medicaid expansion was associated with a statistically significant overall reduction in hazard among adults with lung cancer (DiD HR, 0.88; 95% CI, 0.85–0.91; −12.0% change in hazard) (Table 2). When stratified by race and ethnicity, non-Hispanic White patients experienced a 13.0% relative reduction in CSM (HR, 0.87; 95% CI, 0.83–0.90), and Hispanic patients and non-Hispanic Asian or Pacific Islander (NHAPI) patients had similar or greater improvements (Hispanic HR, 0.88; 95% CI, 0.80–0.97; NHAPI HR, 0.83; 95% CI, 0.71–0.97). In contrast, the DiD estimate among non-Hispanic Black patients was close to the null (HR, 0.98; 95% CI, 0.90–1.07), and the estimate for Native American patients was imprecise with wide confidence intervals spanning substantial benefit and harm (HR, 1.09; 95% CI, 0.68–1.75). These patterns suggest that Medicaid expansion was associated with meaningful improvements in cancer-specific survival overall and for several racial and ethnic groups, but benefits were not uniform across all populations (Table 2).

**Table 2:** Difference-in-Differences Estimates of Cancer-Specific Mortality After Medicaid Expansion, Overall and by Race/Ethnicity.

Race/Ethnicity	DiD HR (95% CI)^a,b^	Change in hazard^c^, %	χ² (df)^d^	P value
All	0.88 (0.85–0.91)	−12.0 (−15.0 to −9.0)	58.76 (1)	<.001
White	0.87 (0.83–0.90)	−13.0 (−17.0 to −10.0)	46.21 (1)	<.001
Black	0.98 (0.90–1.07)	−2.0 (−10.0 to 7.0)	0.20 (1)	.66
Hispanic	0.88 (0.80–0.97)	−12.0 (−20.0 to −3.0)	7.33 (1)	.007
NHAPI	0.83 (0.71–0.97)	−17.0 (−29.0 to −3.0)	5.28 (1)	.02
Native American	1.09 (0.68–1.75)	+9.0 (−32.0 to 75.0)	0.13 (1)	.71

Abbreviations: ACA, Affordable Care Act; DiD, difference-in-differences; HR, hazard ratio; NHAPI, non-Hispanic Asian or Pacific Islander.

^a^HRs estimated from Cox proportional hazards models with an interaction term for expansion status (California vs Texas), time period (pre- vs post-ACA), and race/ethnicity, with robust standard errors.

^b^HRs less than 1.00 indicate greater improvement in cancer-specific mortality after Medicaid expansion in California than in Texas.

^c^Percentage change in hazard calculated as (HR_DiD − 1) × 100.

^d^Joint χ² test from Wald statistics evaluating heterogeneity across racial and ethnic subgroups.

### Overall mortality by race/ethnicity

Patterns for OM closely paralleled those observed for CSM (Table 3). Overall, Medicaid expansion was associated with an 11.0% relative reduction in the hazard of death (DiD HR, 0.89; 95% CI, 0.86–0.91). Non-Hispanic White patients again demonstrated a 13.0% lower hazard of OM after expansion (HR, 0.87; 95% CI, 0.84–0.91), and Hispanic and NHAPI patients experienced 10.0% and 19.0% reductions, respectively (Hispanic HR, 0.90; 95% CI, 0.83–0.98; NHAPI HR, 0.81; 95% CI, 0.70–0.94). In contrast, the DiD estimate remained near null for non-Hispanic Black patients (HR, 0.98; 95% CI, 0.90–1.06) and was highly imprecise for Native American patients. Taken together, these findings indicate that Medicaid expansion was associated with significant reduction in mortality among most racial and ethnic groups, but similar gains were not observed among Black patients (Table 3).

**Table 3:** Difference-in-Differences Estimates of Overall Mortality After Medicaid Expansion, Overall and by Race/Ethnicity.

Race/Ethnicity	DiD HR (95% CI)^a,b^	Change in hazard^c^, %	χ² (df)^d^	P value
All	0.89 (0.86–0.91)	−11.0 (−14.0 to −9.0)	61.51 (1)	<.001
White	0.87 (0.84–0.91)	−13.0 (−16.0 to −9.0)	48.28 (1)	<.001
Black	0.98 (0.90–1.06)	−2.0 (−10.0 to 6.0)	0.27 (1)	.60
Hispanic	0.90 (0.83–0.98)	−10.0 (−17.0 to −2.0)	5.79 (1)	.02
NHAPI	0.81 (0.70–0.94)	−19.0 (−30.0 to −6.0)	7.73 (1)	.005
Native American	0.94 (0.61–1.44)	−6.0 (−39.0 to 44.0)	0.08 (1)	.78

Abbreviations: ACA, Affordable Care Act; DiD, difference-in-differences; HR, hazard ratio; NHAPI, non-Hispanic Asian or Pacific Islander.

^a^HRs estimated from Cox proportional hazards models with an interaction term for expansion status (California vs Texas), time period (pre- vs post-ACA), and race/ethnicity, with robust standard errors.

^b^HRs less than 1.00 indicate greater improvement in overall mortality after Medicaid expansion in California than in Texas.

^c^Percentage change in hazard calculated as (HR_DiD − 1) × 100.

^d^Joint χ² test from Wald statistics evaluating heterogeneity across racial and ethnic subgroups.

### Cancer-specific mortality by disease stage and household income

When examined by disease stage, difference-in-differences estimates for CSM showed small and mostly nonsignificant reductions in hazard for localized and regional disease, with DiD HRs of 0.91 (95% CI, 0.80–1.04) and 0.96 (95% CI, 0.89–1.04), respectively (Table 4). In contrast, patients with distant disease experienced a modest but statistically significant 5.0% reduction in CSM (HR, 0.95; 95% CI, 0.91–1.00), and those with missing stage information had the largest relative improvement (HR, 0.78; 95% CI, 0.66–0.93). By neighborhood household income, there was little evidence of benefit among patients residing in lower-income areas (HR, 0.96; 95% CI, 0.91–1.02), whereas those living in higher-income areas (≥$65,000) experienced a 14.0% reduction in CSM (HR, 0.86; 95% CI, 0.82–0.90). These results suggest significant reductions in CSM after Medicaid expansion were concentrated among patients with more advanced or poorly staged disease and those residing in higher-income communities (Table 4).

**Table 4:** Difference-in-Differences Estimates of Cancer-Specific Mortality After Medicaid Expansion, by Disease Stage, Household Income, and Treatment.

Subgroup	DiD HR (95% CI)^a,b^	Change in hazard^c^, %	χ² (df)^d^	P value
Disease stage				
Localized	0.91 (0.80–1.04)	−9.0 (−20.0 to 4.0)	1.75 (1)	0.19
Regional	0.96 (0.89–1.04)	−4.0 (−11.0 to 4.0)	0.81 (1)	0.37
Distant	0.95 (0.91–1.00)	−5.0 (−9.0 to 0.0)	4.64 (1)	0.03
Missing	0.78 (0.66–0.93)	−22.0 (−34.0 to −7.0)	7.76 (1)	0.005
Household income				
<$65,000	0.96 (0.91–1.02)	−4.0 (−9.0 to 2.0)	1.77 (1)	0.18
≥$65,000	0.86 (0.82–0.90)	−14.0 (−18.0 to −10.0)	38.94 (1)	<.001

Abbreviations: ACA, Affordable Care Act; DiD, difference-in-differences; HR, hazard ratio.

^a^HRs estimated from Cox proportional hazards models with an interaction term for expansion status (California vs Texas), time period (pre- vs post-ACA), and the subgroup variable of interest, with robust standard errors.

^b^HRs less than 1.00 indicate greater improvement in cancer-specific mortality after Medicaid expansion in California than in Texas.

^c^Percentage change in hazard calculated as (HR_DiD − 1) × 100.

^d^Joint χ² tests from Wald statistics were significant for disease stage (χ² = 14.97; df = 4; P = .005) and income (χ² = 40.71; df = 2; P < .001), indicating heterogeneity in DiD effects across subgroups.

### Overall mortality by disease stage and household income

Analyses of OM by disease stage yielded a similar pattern, with modest and nonsignificant reductions among patients with localized or regional disease (HR, 0.94; 95% CI, 0.84–1.04 and HR, 0.95; 95% CI, 0.89–1.02, respectively) (Table 5), and statistically significant improvements among those with distant disease (HR, 0.94; 95% CI, 0.90–0.98) and missing stage (HR, 0.81; 95% CI, 0.70–0.94). When stratified by household income, there was again no clear evidence of benefit for patients in lower-income areas (HR, 0.97; 95% CI, 0.92–1.02), whereas individuals in higher-income areas experienced a 14.0% relative reduction in overall mortality (HR, 0.86; 95% CI, 0.82–0.90). These findings indicate that the mortality reductions associated with Medicaid expansion may be more pronounced among patients with advanced disease and those residing in more affluent neighborhoods (Table 5).

**Table 5:** Difference-in-differences estimates of overall mortality after medicaid expansion, by disease stage, household income, and treatment.

Subgroup	DiD HR (95% CI)^a,b^	Change in hazard^c^, %	χ² (df)^d^	P value
Disease stage				
Localized	0.94 (0.84–1.04)	−6.0 (−16.0 to 4.0)	1.48 (1)	0.22
Regional	0.95 (0.89–1.02)	−5.0 (−11.0 to 2.0)	1.68 (1)	0.19
Distant	0.94 (0.90–0.98)	−6.0 (−10.0 to −2.0)	7.36 (1)	0.007
Missing	0.81 (0.70–0.94)	−19.0 (−30.0 to −6.0)	7.48 (1)	0.006
Household income				
<$65,000	0.97 (0.92–1.02)	−3.0 (−8.0 to 2.0)	1.15 (1)	0.28
≥$65,000	0.86 (0.82–0.90)	−14.0 (−18.0 to −10.0)	45.53 (1)	<.001

Abbreviations: ACA, Affordable Care Act; DiD, difference-in-differences; HR, hazard ratio.

^a^HRs estimated from Cox proportional hazards models with an interaction term for expansion status (California vs Texas), time period (pre- vs post-ACA), and the subgroup variable of interest, with robust standard errors.

^b^HRs less than 1.00 indicate greater improvement in overall mortality after Medicaid expansion in California than in Texas.

^c^Percentage change in hazard calculated as (HR_DiD − 1) × 100.

^d^Joint χ² tests from Wald statistics were significant for disease stage (χ² = 18.00; df = 4; P = .001) and income (χ² = 46.68; df = 2; P < .001), indicating heterogeneity in DiD effects across subgroups.

### Mortality by treatment modality

In treatment-stratified models, Medicaid expansion was associated with consistent reductions in both CSM and OM among patients treated with surgery or chemotherapy (Tables 6–7). For CSM, surgery was associated with a 10.0% lower hazard in California relative to Texas after expansion (DiD HR, 0.90; 95% CI, 0.82–0.98), and chemotherapy was associated with an 8.0% reduction (HR, 0.92; 95% CI, 0.88–0.95) (Table 6). Estimates for OM were identical, indicating parallel benefits for all-cause survival (Table 7). These results suggest that Medicaid expansion was linked to improved outcomes among patients who received definitive cancer-directed treatment, with similar magnitudes of benefit for surgery and chemotherapy.

**Table 6:** Difference‑in‑differences estimates of cancer‑specific mortality after medicaid expansion, by treatment.

Treatment Group	DiD HR (95% CI)^a,b^	Change in hazard^c^, %	χ² (df)^d^	P value
Surgery	0.90 (0.82–0.98)	−10.0 (−18.0 to −2.0)	5.39 (1)	0.02
Chemotherapy	0.92 (0.88–0.95)	−8.0 (−12.0 to −5.0)	20.63 (1)	<.001

Abbreviations: ACA, Affordable Care Act; DiD, difference‑in‑differences; HR, hazard ratio.

^a^HRs estimated from Cox proportional hazards models with interaction terms for expansion status (California vs Texas) and time period (pre‑ vs post‑ACA), with robust standard errors.

^b^Percentage change in hazard calculated as (HR − 1) × 100.

**Table 7:** Difference‑in‑differences estimates of overall mortality after medicaid expansion, by treatment.

Treatment Group	DiD HR (95% CI)^a,b^	Change in hazard^c^, %	χ² (df)^d^	P value
Surgery	0.90 (0.82–0.98)	−10.0 (−18.0 to −2.0)	5.39 (1)	0.02
Chemotherapy	0.92 (0.88–0.95)	−8.0 (−12.0 to −5.0)	20.63 (1)	<.001

Abbreviations: ACA, Affordable Care Act; DiD, difference‑in‑differences; HR, hazard ratio.

^a^HRs estimated from Cox proportional hazards models with interaction terms for expansion status (California vs Texas) and time period (pre‑ vs post‑ACA), with robust standard errors.

^b^Percentage change in hazard calculated as (HR − 1) × 100.

### Assessment of the parallel trends assumption

To evaluate the parallel trends assumption underlying the difference-in-differences analysis, we assessed pre-policy mortality patterns in California and Texas using both graphical and statistical approaches. In the pre–Affordable Care Act period (2007–2013), Kaplan–Meier survival curves showed higher baseline survival in California compared with Texas; however, the trajectories evolved in a largely parallel manner over time. Formal pre-trend testing using Cox proportional hazards models with state-by-year interaction terms restricted to the pre-ACA period demonstrated no evidence of differential mortality trends between states (joint Wald χ² = 2.88; P = 0.82). Together, these findings support the plausibility of the parallel trends assumption. Full results, including Kaplan–Meier curves and interaction estimates, are provided in the Supplement (S1 Fig and S1 Table).

## Discussion

In this population based DiD analysis comparing adults diagnosed with lung cancer in California (a Medicaid expansion state) with those in Texas (a non‑expansion state), Medicaid expansion was associated with consistent reductions in both CSM and OM.

Across the full cohort, Medicaid expansion corresponded to an approximate 12% reduction in CSM and an 11% reduction in OM, indicating a measurable decrease in the hazard of death among patients in California relative to Texas following ACA implementation. These mortality reductions, however, were not uniform across population groups. Improvements were observed among non‑Hispanic White, Hispanic, and Asian/Pacific Islander individuals, while estimates among non‑Hispanic Black patients were near the null. This suggests that longstanding inequities in lung‑cancer outcomes persist despite policy-driven improvements in insurance coverage [26–30]. Estimates for Native American individuals were imprecise due to small sample sizes, limiting definitive conclusions.

Mortality reductions also varied by disease stage and neighborhood income. Patients diagnosed with distant-stage disease exhibited the largest improvements in both CSM and OM. These patterns suggest that advanced disease outcomes may be particularly sensitive to improved insurance coverage [21,31–34], which can reduce delays in diagnostic evaluation, expand access to systemic therapy, and improve palliative care availability [16,35–37]. Individuals residing in higher‑income communities also experienced mortality reductions, whereas those in lower‑income areas exhibited minimal change [31,38]. Because SEER income measures reflect area‑level (county-level) estimates not individual household earnings these results should be interpreted as indicators of community socioeconomic context rather than personal income.

Treatment‑stratified analyses showed parallel findings. Among patients who underwent surgery or received chemotherapy, Medicaid expansion was associated with 8–10% reductions in the hazard of death. These findings may reflect improved access to timely guideline‑concordant therapies, decreased financial barriers, and enhanced continuity of care in expansion states [39,40]. Collectively, these results demonstrate that insurance expansion may influence multiple components of the lung‑cancer care continuum, from early diagnostic pathways to treatment initiation and follow-up care [18,26,41].

The study’s findings align with and extend prior literature showing mortality reductions after Medicaid expansion [16,17,31]. Unlike some earlier studies in which mortality benefits diminished after adjusting for stage [17,31,42], improvements in our population persisted even after accounting for both stage and treatment differences. This suggests that policy-related gains may operate through multiple mechanisms beyond early detection alone including improved treatment access, increased treatment initiation, reduced delays in care, and better supportive and palliative care integration [39,43,44].

### Limitations

Several limitations should be considered. First, SEER does not collect detailed systemic treatment information (e.g., immunotherapy, targeted therapy), comorbidity burden, smoking status, patient-reported outcomes, or baseline insurance status. These omissions limit the ability to adjust for clinically meaningful confounders. Second, income is measured at the area level, introducing ecological misclassification that may attenuate subgroup-specific associations. Third, while lung cancer has relatively short survival times, follow‑up may nonetheless be insufficient to fully assess long‑term mortality effects. Fourth, residual unmeasured confounding and secular trends despite the strengths of a DiD framework cannot be completely excluded.

Despite these limitations, this study provides evidence that Medicaid expansion under the ACA was associated with meaningful reductions in CSM and OM among adults with lung cancer, with the strongest benefits observed among individuals with advanced disease, those receiving active treatment, and residents of higher‑income communities. Persistent absence of improvement among non‑Hispanic Black patients highlights the need for complementary interventions targeting structural inequities in cancer diagnosis, treatment access, and care coordination. Future research should examine mechanisms underlying subgroup differences, evaluate long‑term mortality trajectories, and identify multipronged policy and clinical strategies capable of reducing persistent disparities in lung‑cancer outcomes.

## Conclusion

In summary, Medicaid expansion was associated with measurable reductions in the hazard of cancer-specific and overall mortality among adults with lung cancer, with the greatest benefits observed in patients with advanced disease, those receiving active treatment, and individuals residing in higher-income communities. However, the absence of mortality improvement among non-Hispanic Black patients underscores persistent structural inequities that insurance expansion alone does not resolve. These findings highlight both the potential and the limitations of coverage-focused policy interventions and emphasize the need for targeted, equity-driven strategies to improve lung-cancer outcomes across all population groups.

## Supporting information

S1 File Supplementary Figure and Table. This file contains: (A) Pre-ACA Kaplan–Meier survival curves comparing California and Texas. (B) Pre-ACA parallel trends assessment using state-by-year interactions.
(DOCX)
