# Supplementary material for: Association of medicaid expansion with lung cancer–specific and overall mortality: A difference-in-differences analysis
Source: PLoS One. 2026 Apr 9;21(4):e0332292. doi: 10.1371/journal.pone.0332292 (PMC13065035; doi:10.1371/journal.pone.0332292)
Supplement: S1 File — This file contains: (A) Pre-ACA Kaplan–Meier survival curves comparing California and Texas. (B) Pre-ACA parallel trends assessment using state-by-year interactions. (DOCX) [file pone.0332292.s001.docx]

**S1 File. Supplementary Figure and Table**

**S1 Figure. Pre-ACA Kaplan–Meier survival curves comparing California and Texas**

*Kaplan–Meier survival curves for adults diagnosed with lung cancer in California (red dashed line) and Texas (blue solid line) during the pre–Affordable Care Act period (2007–2013). Survival probabilities differed between states, with higher baseline survival observed in California compared with Texas (log-rank P < 0.001). Despite these baseline differences, the curves follow a largely parallel trajectory over time prior to Medicaid expansion, supporting the plausibility of the parallel trends assumption underlying the difference-in-differences analysis.*

## S1 Table. Pre-ACA Parallel Trends Assessment Using State-by-Year Interactions

| Interaction Term | Hazard Ratio | 95% CI (Lower) | 95% CI (Upper) | P value |
| --- | --- | --- | --- | --- |
| State × 2008 | 0.97 | 0.91 | 1.03 | 0.330 |
| State × 2009 | 1.01 | 0.94 | 1.07 | 0.847 |
| State × 2010 | 0.98 | 0.92 | 1.05 | 0.536 |
| State × 2011 | 0.99 | 0.92 | 1.05 | 0.680 |
| State × 2012 | 0.98 | 0.92 | 1.05 | 0.618 |
| State × 2013 | 1.02 | 0.95 | 1.09 | 0.635 |
| Joint Wald test (State × Year) | — | — | — | P = 0.824 |

*S1 Table shows Cox proportional hazards models restricted to the pre-ACA period (2007–2013), including interaction terms between state (California vs Texas) and diagnosis year. Hazard ratios near unity and non-significant interaction terms indicate no evidence of differential pre-policy mortality trends between states. The joint Wald test evaluates whether all state-by-year interaction terms are simultaneously equal to zero.*

## Parallel Trends Assessment

Assessment of the parallel trends assumption was conducted using both graphical and statistical approaches. Kaplan–Meier survival curves restricted to the pre-ACA period (2007–2013) demonstrated higher baseline survival in California compared with Texas; however, the curves evolved in a largely parallel fashion without evidence of divergence over time. Formal pre-trend testing using Cox regression models with state-by-year interaction terms showed no statistically significant differential trends prior to Medicaid expansion (joint Wald χ² = 2.88, P = 0.82). Together, these findings support the plausibility of the parallel trends assumption underlying the difference-in-differences analysis.
